# The Three Ps: Psychiatry, Pharmacy, and Pharmacogenomics, a Brief Report From New Zealand

**DOI:** 10.3389/fpsyt.2019.00690

**Published:** 2019-09-20

**Authors:** Simran D.S. Maggo, Kyra L.V. Sycamore, Allison L. Miller, Martin A. Kennedy

**Affiliations:** ^1^Gene Structure and Function Lab, Carney Centre for Pharmacogenomics, Department of Pathology & Biomedical Science, University of Otago, Christchurch, New Zealand; ^2^Pharmacy Department, Canterbury District Health Board, Christchurch, New Zealand

**Keywords:** pharmacogenomics, psychiatry, pharmacogenetic testing, *CYP2D6*, *CYP2C19*, antidepressant

## Abstract

We describe a case series of 22 individuals who were referred to our laboratory by a pharmacist based in a mental health hospital, for pharmacogenetic analysis due to severe or unexpected adverse drug reactions (ADRs) to psychiatric medication. The participants were genotyped for common variation in the *CYP2D6*, *CYP2C19*, and *CYP2C9* genes, using Sanger sequencing. We tested variants in these genes as they have the strongest evidence with respect to altering the pharmacokinetics of commonly prescribed psychiatric medicine. Looking specifically at the subset of 18 European study participants, we observed a comparatively high but non-significant rate of pharmacogenetic variants, compared to allele frequency surveys in unselected population samples. For *CYP2D6*, we observed an elevated frequency of both poor (17%) and intermediate (33%) metabolizers when compared with previously reported frequencies (6% and 12% respectively). For *CYP2C19*, we observed an increased frequency of intermediate (33%) and ultra-rapid (17%) metabolizers compared to expected frequencies (21% and 4% respectively). For *CYP2C9*, the frequency of intermediate metabolizers (22%) was elevated compared to the expected population frequency (11%). While sample size is a major limitation of this brief report, we can conclude that patients with adverse reactions to antidepressant or antipsychotic drugs selected by a specialist mental health pharmacist appear to have a relatively high rate of genetic variants in pharmacogenes known to affect the pharmacokinetics of these drugs. The selective application of such pharmacogenetic tests by clinical pharmacists may be a valuable approach to clarify the basis for adverse or unusual responses to medication, and to guide ongoing prescribing decisions for this group of patients.

## Introduction

The study of how genetic variability affects individual drug metabolism and drug response is defined as pharmacogenomics (1). Individual response to prescribed medication and/or drug-induced adverse drug reactions (ADRs) has been a field of ongoing investigation for the last three decades (1). The United States Food and Drug Administration (US FDA) lists over 150 drugs with pharmacogenomic information related to drug safety on their labeling (2). Of these, approximately 15% of listed pharmacogenomic markers fall under the medical field of psychiatry and highlight the “biomarker” as *CYP2D6* and/or *CYP2C19* (2). These two cytochrome P450 (CYP450) enzymes, particularly CYP2D6, are encoded by highly polymorphic genes, which have been reported to influence individual response and associated ADRs to prescribed drugs not only in psychiatry, but to a wide range of pharmacotherapy (3–5).

Although such variation contributes to marked differences in drug efficacy and predisposes to ADRs, it has proven challenging to incorporate pharmacogenetic tests into clinical practice (1, 6–10). Many factors contribute to this situation, including lack of guidelines around implementation of test results, limited clinician awareness of tests, and lack of evidence for efficacy in a range of clinical settings. The Clinical Pharmacogenetic Implementation Consortium (CPIC) is effectively addressing the question of implementation guidelines (6). With respect to psychiatry, peer-reviewed CPIC guidelines exist for selective serotonin reuptake inhibitors (SSRIs) (11) and tricyclic antidepressants (TCAs) (12). The CPIC guidelines advise on dosing changes for known polymorphisms in the *CYP2D6* and/or *CYP2C19* genes. These polymorphisms, often referred to as single nucleotide polymorphisms (SNPs), can influence inter-individual pharmacokinetics, drug response, and efficacy, and increase the risk of ADRs. *CYP2D6* and *CYP2C19* genes are highly polymorphic, with over 150 known variants collectively (13). Given a patient’s genotype for *CYP2D6* and/or *CYP2C19*, it is possible to use CPIC’s curated list of known variants defined by their reference sequence (rs) number and/or star (*) terminology to interpret a clinically relevant action to take with respect to drug dosing or choosing an alternative drug treatment (11).

Clinician awareness of tests is an important factor in uptake of pharmacogenetics, and many arguments have been made in support of pharmacists as a well-trained, knowledgeable, and appropriate professional group for guiding the uptake of pharmacogenetics (14, 15). As a professional group, pharmacists are willing and eager to use pharmacogenetic information as part of medication management and review for these problematic cases. The role of pharmacists as key professionals in implementation of pharmacogenetic tests has been proposed and explored in several overseas studies (16–19). Many large pharmacogenetic initiatives are driven by, or depend upon, pharmacists to work with patients and doctors, requesting tests, interpreting results, and providing education (19, 20). At least five major USA medical centers have implemented pharmacogenetic analysis into clinical care programs (9, 21), and many of these involve expert input by pharmacists. The Ubiquitous Pharmacogenomics (U-PGx) Consortium, a collaborative effort across 16 different organizations in 10 European countries, involves pharmacists and is working toward implementation of pharmacogenetics-informed prescribing into patient care (7, 22, 23). Pharmacists are also significant contributors to the activities of CPIC (19).

Developing an evidence base for the value of pharmacogenetics in particular settings is important to guide future implementation decisions. Initial reports describe potential clinical settings in which pharmacists can help to implement pharmacogenetic testing (18, 24). Preemptive testing prior to drug administration is logistically difficult and the cost-effectiveness of implementing pharmacogenetic tests for all patients prior to prescribing specific drugs is still unclear (10, 24–28). However, in the mental health setting, patients are often suffering from chronic disorders that can be difficult to treat, and pharmacogenetics may offer additional information to improve management of those patients experiencing ADRs or poor responses. Targeting pharmacogenetic analysis to this subgroup of patients may provide information that improves treatment outcomes and potentially reduces costs.

In this report, we describe a case series of 22 patients referred to us for pharmacogenetic testing by a specialist mental health pharmacist working in a hospital-based mental health setting. All patients were reported as having ADRs to antidepressant and/or antipsychotic therapy at standard or low doses and were selected on the expectation that pharmacogenetic analysis would provide additional information to guide ongoing treatment decisions.

## Methods

### Participants

The pharmacist had sole discretion in the selection and referral of participants who were deemed to have serious and/or unusual ADRs to antidepressant or antipsychotic medication. Side effects to antidepressant and antipsychotic medications are common and sometimes even expected after initiation or dose changes. For this study, the majority of patients who were referred had experienced a side effect that was particularly severe or unexpected, having taken other medicine and patient-related factors into account. Other patients were referred due to a lack of response to a medicine where despite being on a high dose, there was a lack of clinical effect and an unexpected absence of ADRs.

Referred patients were then recruited into the ongoing Understanding Adverse Drug Reactions Using Genomic Sequencing (UDRUGS) (27, 28) study. This study is approved by the Southern Health and Disability Ethics Committee, New Zealand (ethics approval number URA/11/11/065). After informed consent was received from the patient and/or guardian (in case of patients under the age of 16), three tubes of blood in lavender tubes ethylenediaminetetraacetic acid (EDTA) were obtained, for extraction of DNA and subsequent genetic analysis. Where blood samples were not available (due to a patient living in a rural or distant location), a saliva sample was acquired using an Oragene collection kit (DNA Genotek, Ottawa, ON, Canada). All samples were anonymized and assigned a UDRUGS code.

For each patient, the pharmacist also completed a questionnaire to document ADRs, as well as participants’ demographics, disease and medication histories, a detailed account of the ADR(s), and objective data (if any).

### Genetic Analysis

Genomic DNA was extracted from peripheral blood using a method modified from Miller et al. (29). This protocol consists of a general salting-out method followed by a phenol–chloroform purification step. DNA extraction from saliva was carried out according to the manufacturer’s instructions (Oragene OG-250 kit; DNA Genotek, ON, Canada). Protocol: http://www.dnagenotek.com/US/pdf/PD-PR-006.pdf.

Genetic analysis was conducted by Sanger sequencing for common variants in *CYP2D6*, *CYP2C9*, and *CYP2C19* genes. *CYP2D6* genotyping was conducted using a two-stage polymerase chain reaction (PCR). The first-step PCR reaction was conducted using a previously described method (30) to isolate a PCR product approximately 6.6 kb in length. This step isolates a *CYP2D6* product from a neighboring *CYP2D7* pseudogene. This step also allows the identification of *CYP2D6* duplication or deletion alleles using specific primers. In brief, the initial PCR consisted of a 10 µl reaction, which was set up as follows: 1× KAPA- Long Range (LR) reaction buffer (Kapa Biosystems, Wilmington, USA), 1.75 mM Mg^2+^, 0.3 mM of each deoxyribonucleotide triphosphate (dNTP), 0.4 µM of each 6.6 kb primer, 0.3 µM of duplication or deletion primers, 1 M betaine, 0.25 U of KAPA LR DNA (Kapa Biosystems, Wilmington, USA) polymerase, and 50 ng of DNA. Cycling conditions included initial heating to 94°C for 3 min, followed by 35 cycles of 94°C for 25 s, 68°C for 10 s, and 68°C for 7 min, and a final elongation step of 72°C for 7 min. Four microliters of the initial PCR product was run on a 1% agarose gel to confirm the successful amplification of a 6.6 kb product and identification of any 3.5 kb duplication or deletion bands. Duplication or deletion alleles were confirmed by comparison with in-house controls (known samples with *CYP2D6* duplication or deletion). No duplications or deletions were identified in the current cohort of 22 participants. The second stage of the PCR utilized a 1,000-fold diluted PCR product (6.6 kb PCR product from first stage) to conduct a nested PCR to cover the *CYP2D6* gene. For the second stage, a standard 20 µl reaction was set up as follows: 1× Fisher Biotec reaction buffer, 2.5 ng/µl template, 0.2 mM of each dNTP, 0.2 µM of each forward and reverse primer, 1.5 mM Mg^2+^, and 0.025 U/µl of TAQ-TI DNA polymerase (Fisher Biotec, Wembley, WA, Australia). This method allows for the identification of common variants referred to as *2, *3, *4, *5 (deletion), *6, *7, *8, *9, *10, and *41, as well as other variants not often assessed in commonly available assay kits.

*CYP2C19* and *CYP2C9* genotyping was conducted using a total of 12 primer pairs to assess common variants known to affect drug pharmacokinetics. A 20 µl PCR reaction was set up as described above with *CYP2C19*-specific primers. Cycling conditions were as follows: initial heating to 94 for 2 min; followed by 15 cycles of 94°C for 15 s, 65°C for 15 s (reducing by 1 degree per cycle), and 72°C for 1 min; followed by 20 cycles of 94°C for 15 s, 50°C for 15 s, and 72°C for 1 min.

After confirmation of PCR products on a 1% agarose gel, PCR products were prepared for bi-directional Sanger sequencing, which was carried out on an Applied Biosystems 3130*xl* genetic analyzer for capillary electrophoresis. Bioinformatic analysis of generated sequences was done with the software Geneious V8.1.9 (Biomatters, Ltd., Auckland, New Zealand). In brief, generated sequencing files were aligned against the reference gene sequences from the National Center for Biotechnology Information (NCBI) (https://www.ncbi.nlm.nih.gov/gene) of *CYP2D6* (ID: 1565), *CYP2C19* (ID: 1557), and *CYP2C9* (ID: 1559). These reference gene sequences were annotated with variants from the Pharmacogene Variation (PharmVar) Consortium (http://www.pharmvar.org) (13). Participant samples were assigned “star” genotypes using translation tables available on PharmGKB. For example the *CYP2D6* translation table is available here: https://www.pharmgkb.org/gene/PA128/haplotype (31).

CYP2D6, CYP2C9 and CYP2C19 metabolizer status was assigned using CPIC guidelines (32)

### Statistical Analysis

As our cohort comprised 18 European and 4 Māori participants, we only conducted statistical comparisons using data from the 18 European participants. The reasons for this were that accurate allele frequency and phenotype data are not available for large samples of Māori, and we only had four Māori participants. Predicted phenotype frequencies (referred to as observed) of 18 European study participants for the three genes were compared to those reported by Mostafa et al. (33) (referred to as expected) in a comparable Oceanic-European population. We conducted a comparison of proportions test in the program MedCalc (Version 19.0.4, MedCalc Software bvba, Ostend, Belgium; free trial license). This utilizes an “N − 1” chi-squared test (34).

## Results

A total of 22 study participants (3 males and 19 females) were referred and accepted into the UDRUGS study. Participant demographics, brief mental health medication history, ADR genotype, and predicted phenotype are shown in **Table 1**.

**Table 1:** Participant demographics, genotype, and predicted phenotype.

Participant ID	Sex	Ethnicity	Smoker	Alcohol	Primary diagnosis	Reason for recruitment	CYP2D6	CYP2C19	CYP2C9
**PT1**	F	European	Yes	Yes	Anorexia, depression/anxiety	Venlafaxine-: night sweats Sertraline: tremor, anxiety, diarrhea Codeine: ineffective	PM (*4/*4)	NM (*1/*1)	NM (*1/*1)
**PT2**	M	European	No	No	Anxiety	Risperidone: dystonia, unstable blood pressure, tingling sensation in extremities, dizziness, tinnitus	IM (*1/*4)	NM (*1/*1)	NM (*1/*1)
**PT3**	F	European	No	No	Anxiety	Fluoxetine: paresthesia, whole-body shakes, insomnia	PM (*4/*4)	RM (*1/*17)	NM (*1/*1)
**PT4**	F	European	No	No	Anxiety, OCD	Fluoxetine: increased anxiety, disturbed sleep	NM (*1/*2)	UM (*17/*17)	NM (*1/*1)
**PT5**	F	European	No	Yes	Anxiety, OCD, psychosis	Clozapine: seizures Risperidone: EPSEs	NM (*1/*2)	IM (*1/*2)	NM (*1/*1)
**PT6**	F	European	No	No	Anorexia	Fluoxetine + olanzapine: muscle rigidity, cogwheeling, increased anxiety	NM (*1/*2)	RM (*1/*17)	NM (*1/*1)
**PT7**	F	European	No	No	Anxiety	Ziprasidone + sertraline: tremor, teeth chattering, tachycardia, seizures Mirtazapine: excessive weight gain Quetiapine: excessive sedation Fluoxetine: suicidal ideation	IM (*1/*4)	UM (*17/*17)	NM (*1/*1)
**PT8**	M	European	No	Yes	Depression	Sertraline + quetiapine: disinhibition, “feeling drunk,” irritability, muscle rigidity	IM (*4/*41)	IM (*1/*2)	IM (*1/*2)
**PT9**	F	European	No	Yes	Depression/anxiety	Sertraline: tremor Fluoxetine: increased anxiety	PM (*4/*4)	NM (*1/*1)	NM (*1/*1)
**PT10**	F	European	No	Yes	Depression	Fluoxetine: numb feeling Codeine: ineffective	IM (*1/*4)	NM (*1/*1)	NM (*1/*1)
**PT11**	F	NZ Māori	No	No	Anxiety	Citalopram: increased drowsiness, increased anxiety	NM (*2/*41)	RM (*1/*17)	NM (*1/*1)
**PT12**	F	European	No	Yes	Depression/anxiety	Mirtazapine: aggression Nortriptyline: tremor, diaphoresis, decreased libido Venlafaxine: decreased appetite, decreased libido Quetiapine: “foggy brain”	IM (*9/*41)	IM (*1/*2)	NM (*1/*1)
**PT13**	F	Cook Island Māori	No	No	Depression/anxiety	Quetiapine: dizziness, blurred vision, headaches Escitalopram: ineffective	IM (*4/*41)	IM (*1/*2)	NM (*1/*1)
**PT14**	M	European	No	No	Anxiety	Fluoxetine: ineffective	IM (*1/*4)	IM (*1/*2)	NM (*1/*1)
**PT15**	F	European	No	Yes	Anxiety	Citalopram: tiredness, nausea, nightmares	IM (*1/*4)	UM (*17/*17)	NM (*1/*1)
**PT16**	M	European	Yes	Yes	ADHD, depression	Methylphenidate: psychosis, agitation, aggression Fluoxetine: low mood, aggression	NM (*1/*10)	IM (*1/*2)	NM (*1/*1)
**PT17**	F	European	No	No	Depression	Fluoxetine: suicidal ideation, decreased concentration Moclobemide: decreased mood when dose increased	NM (*1/*2)	IM (*1/*2)	IM (*1/*2)
**PT18**	F	European	Yes	Yes	Depression	Fluoxetine: ineffective Venlafaxine: headaches Mirtazapine: weight gain, sleep paralysis Bupropion: pain in hands/feet	NM (*1/*1)	RM (*1/*17)	IM (*1/*2)
**PT19**	F	European	No	No	OCD	Clomipramine: ineffective (and unexpectedly low plasma concentrations)	NM (*1/*41)	NM (*1/*1)	NM (*1/*1)
**PT20**	F	NZ Māori	No	No	Depression/anxiety	Sertraline: vomiting when dose increased over 37.5 mg/day Quetiapine: excessive sedation	NM (*1/*1)	PM (*2/*2)	NM (*1/*1)
**PT21**	M	NZ Māori	Yes	Yes	Psychosis	Risperidone: dystonia, EPSEs Olanzapine: severe sedation.	NM (*1/*1)	NM (*1/*1)	IM (*1/*3)
**PT22**	F	European	No	No	Depression/anxiety	Fluoxetine: tremor in hands and legs, serotonin syndrome Sertraline: similar to fluoxetine	NM (*1/*2)	RM (*1/*17)	IM (*1/*3)

NM, normal metabolizer; IM, intermediate metabolizer; PM, poor metabolizer; RM, rapid metabolizer; UM, ultra-rapid metabolizer; OCD, Obsessive compulsive disorder; ADHD, Attention Deficit Hyperactivity Disorder; EPSE, Extrapyramidal Side Effects. Genotype is derived from (13).

**Figure 1:**
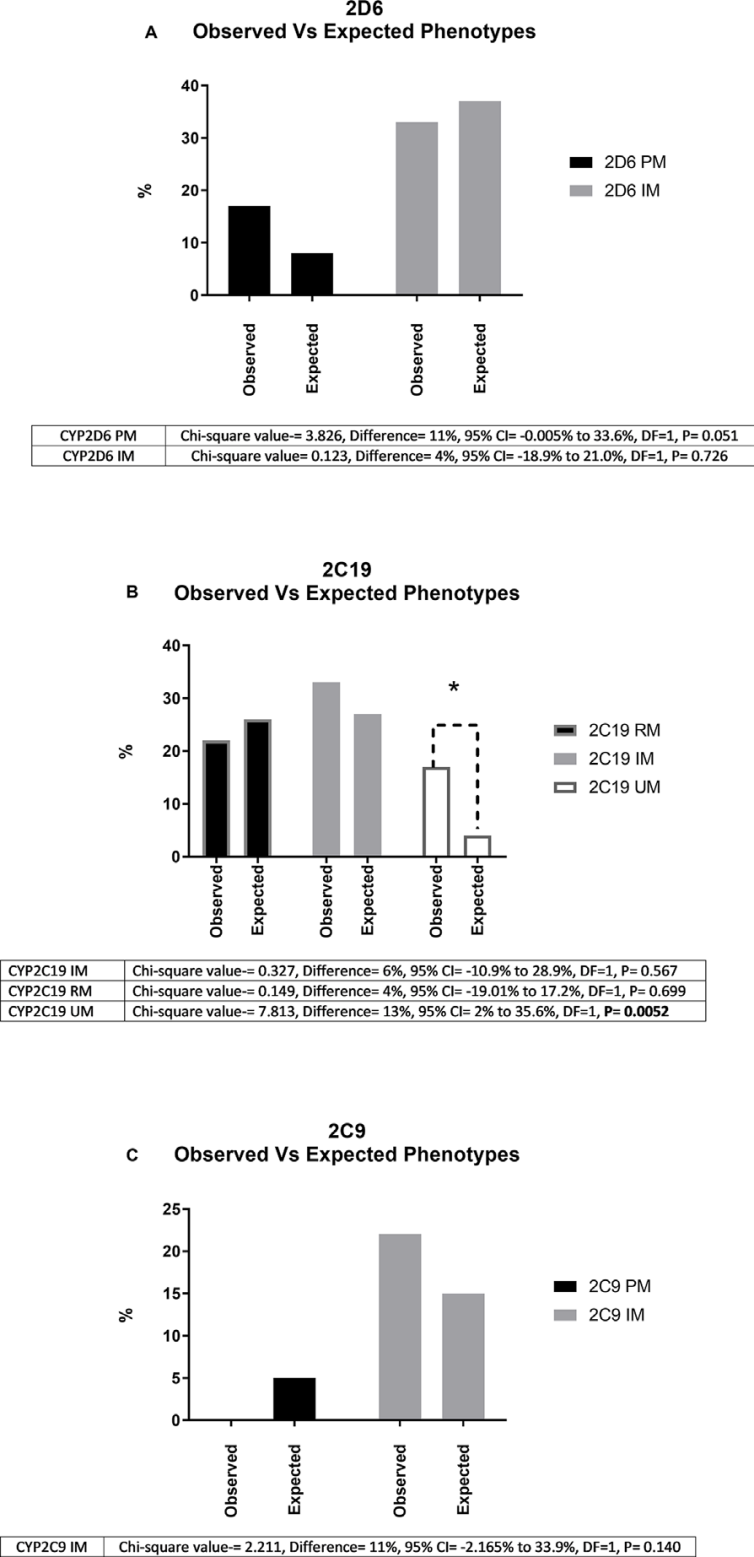
Shows observed and expected frequencies (%) of phenotypes for the three genes **(A**, *CYP2D6*; **B**, *CYP2C19*; **C**, *CYP2C9***)** investigated for 18 study participants of European descent. “Observed” indicates the % of poor (PM), intermediate (IM), rapid (RM), and ultra-rapid (UM) metabolizers in our cohort. “Expected” indicates the corresponding frequencies reported in a recent report of over 5,000 unselected patients in a comparable Oceania population. * Indicates significantly different (P < 0.05) using chi-squared analysis. As shown in **Figure 1(B)**, for *CYP2C19*, we did not encounter any European PMs from a total of 18 European cases. We did observe a *CYP2C19* PM in one out of four Māori study participants (**Table 1**).

Phenotyping and genotyping summary results for these participants across a range of key variants are shown in **Tables 2** and **3**, respectively. We compared the genotype frequencies of the 18 European and 4 Māori participants of this group with expected frequencies for a largely European sample based on data from gnomAD and a recent publication reporting allele frequencies from Australia (33, 35). As previously mentioned, our cohort only included four M*ā*ori participants, and accurate allele frequency data from large data sets is not currently available for M*ā*ori. While we have included these results for clarity, statistical comparisons are not possible. Looking specifically at the 18 European participants, this comparison suggested that the case cohort had an increased frequency of the null function *4 allele in CYP2D6 when compared to expected allele frequencies from the gnomAD database and a recent publication by Mostafa et al. (33) (**Table 2**). We did not observe a similar trend for CYP2C19 or CYP2C9. When we categorized the 18 European participants into phenotypes according to Caudle et al. (32) (**Figure 1**), the observed frequency of CYP2C19 ultra-rapid metabolizers (UMs) was significantly (chi-squared value = 7.813, difference = 13%, 95% CI = 2% to 35.6%, DF = 1, P = 0.0052) elevated when compared with the expected frequency (**Figure 1B**). Similarly, we noted a trend of increased frequency of poor and intermediate metabolizers (PMs and IMs) of CYP2D6 (**Figure 1A**) and IMs for CYP2C19 (**Figure 1B**), although neither of these results was statistically significant (**Figures 1A-C**).

**Table 2:** Predicted phenotype summary for 22 study participants, broken down by ethnicity (E = European (n = 18), M = Māori (n = 4)).

Gene	Normal metabolizers (NMs)	Intermediate metabolizers (IMs)	Poor metabolizers (PMs)	Rapid metabolizers (RMs)	Ultra-rapid metabolizers (UMs)
CYP2D6	E = 8 (44%) M = 3 (75%)	E = 7 (39%) M = 1 (25%)	E = 3 (17%) M = 0		E = 0 M = 0
CYP2C19	E = 6 (33%) M = 1 (25%)	E = 6 (33%) M = 1 (25%)	E = 0 M = 1 (25%)	E = 3 (17%) M = 1 (25%)	E = 3 (17%) M = 0
CYP2C9	E = 14 (78%) M = 3 (75%)	E = 4 (22%) M = 1 (25%)	E = 0 M = 0		

**Table 3:** Genotyping information for 22 study participants, broken down by ethnicity [E = European (n = 18), M = Māori (n = 4)].

Gene	Star allele and (rsID)	Cohort allele frequency by ethnicity (%)	gnomAD allele frequency^#^ (%) (35)	Mostafa et al. (33) Allele frequency (%) (33)
**CYP2D6** ^a^	*4 (rs3892097)	E = 33 M = 25	19.6	17.8
	*9 (rs5030656)	E = 3 M = 0	2.6	2.3
	*10 (rs1065852)	E = 3 M = 0	21.6	3.3
	*41 (rs28371725)	E = 6 M = 25	9.3	10.2
**CYP2C19**^b^	*2 (rs4244285)	E = 16.7 M = 37.5	14.7	16.4
	*17 (rs12248560)	E = 27.8 M = 12.5	23	20.2
**CYP2C9**	*2 (rs1799853)	E = 8.3 M = 0	12.6	12.8
	*3 (rs1057910)	E = 0 M = 12.5	6.8	6.9

^#^gnomAD allele frequency is for non-Finnish Europeans.

a CYP2D6 variants *3, *5, *6, *7, *8, *11, and *12 were also tested. None of the 22 patients in this case series expressed these variants.

b CYP2C19 variants *3, *4, *5, *6, *7, and *8 were also tested. None of the 22 patients in this case series expressed these variants.

## Discussion

This brief report describes a series of 22 patients who had unusual or severe ADRs to antidepressant and/or antipsychotic medication, selected and referred to us by a specialist mental health pharmacist working in a hospital. These patients were enrolled into the UDRUGS study to enable analysis of three pharmacogenes. Genotyping of the *CYP2D6*, *CYP2C19*, and *CYP2C9* genes showed significantly increased frequency of some variants that have been reported to alter the pharmacokinetics of antidepressant or antipsychotic medications. In particular, we observed an elevated number of *CYP2D6* IMs and PMs, *CYP2C19* IMs and UMs, and *CYP2C9* IMs.

Recently, Bousman and colleagues published a research paper suggesting a “minimum evidence based genetic testing panel” for psychiatry (36). This panel consists of five genes, *CYP2D6*, *CYP2C19*, *CYP2C9*, *HLA-A*, and *HLA-B*. Like Bousman and colleagues, we included the three CYP genes and 14 variants in these genes. We decided to exclude *HLA-A* and *HLA-B* from our testing panel, as we did not have any study participants with ADRs to carbamazepine, oxcarbazepine, or phenytoin. Based on this exclusion, we could have also excluded *CYP2C9* but decided to keep it, as the enzyme CYP2C9 is a minor metabolism pathway for several antidepressants.

With respect to our findings for *CYP2D6*, we observed an increased frequency of *CYP2D6* IMs (33%) and PMs (17%) compared to what would be expected in individuals of European descent. Specifically, for a New Zealand European population, a previous investigation carried out by Roberts et al. (37) reported 10 individuals with a *CYP2D6* PM genotype out of a total cohort of 125 patients (8%) (37). The current cohort of patients is significantly smaller when compared with Roberts et al. (37); however, larger studies and two meta-analyses all report the frequency of *CYP2D6* PMs in a Caucasian population to be approximately 5% (38–41). Also, it is reported that *CYP2D6* PMs are at an increased risk of antidepressant-induced ADRs as a result of reduced metabolism of the parent drug, in this case, venlafaxine (42). In a similar report by Rau et al. (43), the frequency of PMs was 29% (8/25) in a cohort of 28 German patients selected to be genotyped based on having an ADR to antidepressant drug(s) (43). The authors further concluded that the frequency of PMs in the ADR group was fourfold greater than the average number of *CYP2D6* PMs (7%) in the German population (43). Similarly, reports with other antidepressant or antipsychotic medication such as risperidone have reported that patients who had a moderate ADR or discontinued risperidone therapy due to an ADR had an odds ratio (OR) of 3.0 and 3.1 of being a *CYP2D6* PM, respectively (44). Furthermore, a study of other variables using logistic regression models (co-prescription of interacting drugs or drug metabolite levels) identified *CYP2D6* PM status as the only significant variable that predicted ADRs while taking risperidone or discontinuation of risperidone due to ADRs (44).

The STAR*D (Sequenced Treatment Alternatives to Relieve Depression) trial is one of the largest studies to assess the effectiveness of depression treatments in patients diagnosed with major depressive disorder (45). Peters et al. (46) utilized a cohort from the STAR*D cohort, who had consented to DNA testing. Genetic polymorphisms that may affect citalopram response and tolerance were assessed by sequencing key genetic variants impacting on the pharmacokinetics of citalopram therapy (46). Of the five genes assessed, common variants in the *CYP2D6* and *CYP2C19* genes were analyzed. The authors of this study utilized a two-stage design to reduce type I error. In the discovery cohort, seven variants from five genes were identified as significantly affecting either response or tolerability to citalopram. Of particular relevance to this article are the *4 and *5 variants in the *CYP2D6* gene and the *2 variant in the *CYP2C19* gene, all of which result in reduced or null function of the enzyme. However, the association of these variants with response and/or tolerability to citalopram was not replicated in the validation cohort (46).

Comparatively, a study carried out in 196 patients who were part of the GENDEP (Genome-Based Therapeutic Drugs for Depression) study found a significant association between *CYP2C19* and *CYP2D6* genotype and steady state escitalopram concentrations (47). Escitalopram is the S-enantiomer of citalopram, which in comparison is a racemic mixture of the S- and R-enantiomers. Compared with *CYP2D6* normal metabolizers (NMs), patients genotyped as IM or PM for *CYP2D6* had a higher mean serum escitalopram concentration, but no effect was observed with respect to N-desmethyl escitalopram concentrations (47). For *CYP2C19*, compared with the NMs, patients genotyped as UMs or PMs had significantly different escitalopram serum concentrations. Similar to *CYP2D6*, there was no influence of *CYP2C19* genotype on N-desmethyl escitalopram concentrations (47). This study did not assess ADRs associated with genotype but showed a significant correlation between genotype and serum escitalopram concentrations. Focusing on *CYP2C19* and citalopram or escitalopram in children, Aldrich and colleagues have recently reported that out of 248 inpatients recruited into their study, 237 (95.6%) had at least one side effect that was significantly (p < 0.05) associated with *CYP2C19* IM or PM status. In addition, *CYP2C19* IMs and PMs were reported to be significantly (p = 0.007) more likely to discontinue citalopram/escitalopram therapy when compared with NMs (48).

Limitations to this study are threefold. First, we genotyped common variants in three genes known to be associated with variable drug pharmacokinetics. It is possible that some of the patients could harbor novel variants within either the genes we studied or other genes, which may affect the variability in response to the prescribed medication. We have previously shown that when whole gene analysis is performed, novel variants predicted to affect drug metabolism can be identified (49); however, this is an expensive and time-consuming exercise, which is not possible for all participant samples. Second, our case cohort is small (n = 22), and we only included 18 European study participants in the statistical analysis. This limits our ability to draw strong conclusions and increases the statistical chance of a type II error. Third, we compared observed rates of pharmacogenetic variants in our case cohort with published allele frequencies, drawn from populations of similar ethnic (Oceanic-European) makeup.

In conclusion, the data we obtained suggest that pharmacist identification and referral of mental health patients who are experiencing adverse reactions or unusual responses to their medication can yield a high rate of gene variants likely to explain these poor treatment outcomes. These observations suggest that providing pharmacogenetic data for such selected patients, in collaboration with a hospital pharmacist, is a potentially valuable approach. This approach warrants a much larger study that includes detailed assessment of effects on treatment outcomes and an evaluation of the cost-effectiveness of such an approach.

## Data Availability

All datasets generated for this study are included in the manuscript.

## Ethics Statement

This study was approved by the New Zealand Health and Disability Ethics Committee (HDEC). All participants provided written informed consent to participate in the UDRUGS study. For participants aged 11-15, written informed consent was provided by the participant's legal guardian/next of kin.

## Author Contributions

SM is the main contact for the UDRUGS study. He has been responsible for all genetic testing and reporting back to the specialist mental health pharmacist. SM wrote the first draft of this article. KS was responsible for interpreting the results and reporting back to patients and doctors. KS was responsible for recruitment and consent of the 22 patients described in this case series. KS aided in the proofing of this document. AM provided technical support with respect to DNA extraction and PCR and aided in the analysis of Sanger sequencing results. MK aided in the editing of this manuscript, specifically, the discussion and conclusions.

## Funding

SM’s salary is co-funded by the Jim and Mary Carney Charitable trust and a project grant from the New Zealand Lottery Board.

## Conflict of Interest Statement

The authors declare that the research was conducted in the absence of any commercial or financial relationships that could be construed as a potential conflict of interest.
